# Longrange PCR-based next-generation sequencing in pharmacokinetics and pharmacodynamics study of propofol among patients under general anaesthesia

**DOI:** 10.1038/s41598-017-15657-2

**Published:** 2017-11-13

**Authors:** Oliwia Zakerska-Banaszak, Marzena Skrzypczak-Zielinska, Barbara Tamowicz, Adam Mikstacki, Michal Walczak, Michal Prendecki, Jolanta Dorszewska, Agnieszka Pollak, Urszula Lechowicz, Monika Oldak, Kinga Huminska-Lisowska, Marta Molinska-Glura, Marlena Szalata, Ryszard Slomski

**Affiliations:** 1 Institute of Human Genetics, Polish Academy of Sciences, Strzeszynska 32, 60-479 Poznan, Poland; 2 The NanoBioMedical Centre, Adam Mickiewicz University, Umultowska 85, 61-614 Poznan, Poland; 3 Department of Biochemistry and Biotechnology, University of Life Sciences, Dojazd 11, 60-637 Poznan, Poland; 4 Faculty of Health Sciences, Poznan University of Medical Sciences, Smoluchowskiego 11, 60-179 Poznan, Poland; 5 Laboratory of Neurobiology, Poznan University of Medical Sciences, Przybyszewskiego 49, 60-355 Poznan, Poland; 6 Department of Genetics, Institute of Physiology and Pathology of Hearing, Mokra 17, Warsaw/Kajetany, 05-830 Nadarzyn, Poland; 7 Faculty of Physical Education, Gdansk University of Physical Education and Sport, Kazimierza Gorskiego 1, 80-336 Gdansk, Poland; 8 Department of Computer Science and Statistics, Poznan University of Medical Sciences, Dabrowskiego 79, 60-529 Poznan, Poland; 9 Department of Anaesthesiology and Intensive Therapy, Regional Hospital, Juraszow 7/19, 60-479 Poznan, Poland

## Abstract

The individual response of patients to propofol results from the influence of genetic factors. However, the state of knowledge in this matter still remains insufficient. The aim of our study was to determine genetic predictors of variable pharmacokinetics and pharmacodynamics of propofol within selected 9 genes coding for propofol biotransformation enzymes, receptors and transporters. Our studies are the first extensive pharmaocgenetics research of propofol using high throughput sequencing technology. After the design and optimization of long range PCR-based next-generation sequencing experiment, we screened promoter and coding sequences of all genes analyzed among 87 Polish patients undergoing general anaesthesia with propofol. Initially we found that two variants, c.516 G > T in the *CYP2B6* gene and c.2677 T > G in the *ABCB1* gene, significantly correlate with propofol’s metabolic profile, however after Bonferroni correction the P-values were not statistically significant. Our results suggest, that variants within the *CYP2B6* and *ABCB1* genes correlate stronger with propofol’s metabolic profile compared to other 7 genes. *CYP2B6* and *ABCB1* variants can play a potentially important role in response to this anaesthetic and they are promising object for further studies.

## Introduction

Propofol (2,6-diisopropylphenol)‘s favorable pharmacological properties make it one of the safest and most widely used anaesthetics for intravenous general anaesthesia. However, a large interindividual variability of its pharmacodynamics (PD) and pharmacokinetics (PK) has been reported, which may lead to unexpected side effects^1^. It is known that the biotransformation pathway of propofol includes the action of numerous enzymes, whose polymorphic character may contribute to the individual response of patients to this anaesthetic^2,3^. Moreover, propofol transporting and receptor protein changes, which have a genetic background, could modify propofol action^4,5^. So far, the significance of only few variants of single genes in the propofol biotransformation rate and its dosage has been investigated^6–8^. Unfortunately, these data are often inconclusive. In addition, there are no studies on polygenic analysis in the context of variable PK and PD of propofol. Next-generation sequencing (NGS) technology has become currently an essential tool in extensive pharmacogenetic research, giving the possibility for in-depth analysis of targeted genes’ panel of interest.

The greatest contribution to propofol’s individual response is seen in the variants of the *CYP2C9*, *CYP2B6*, *UGT1A9*, *SULT1A1*, *NQO1* genes, involved in the biotransformation pathway of the anaesthestic and also of transporting proteins: especially P-glycoprotein and serum albumin, encoded by the *ABCB1* and *ALB* genes, as well as the receptor genes *GABRA1* and *ADRA1A* ^4,9,10^. The crucial role can be probably played by three of the genes mentioned above: *CYP2B6* (OMIM123930), *CYP2C9* (OMIM601130) and *UGT1A9* (OMIM606434), coding for metabolizing enzymes: cytochromes P-450 2B6 and 2C9, and UDP-glucuronosyltransferase 1A9, acting mainly in the liver.

The first step in propofol biotransformation is the hydroxylation process conducted mainly by CYP2B6 and CYP2C9 cytochromes, encoded by genes the *CYP2B6* and *CYP2C9*, whose highly polymorphic character contributes to the variable expression level of these proteins and metabolism efficiency in the liver. For instance, two common allelic variants p.Q172H and p.K262R in the *CYP2B6* gene are linked with decreased and increased expression, respectively^11^. For the *CYP2C9* gene, two non-synonymous changes p.R144C (CYP2C9*2) and p.I359L (CYP2C9*3) associated with poor metabolizing phenotypes are a crucial subject of global pharmacogenetic studies^12^. Also the polymorphic gene *UGT1A9* is described as an important pharmacogene due to the involvement in glucuronidation process of many important drugs besides propofol, for example, irinotecan and flavopiridol^13^. Genetic variants of the *UGT1A9* gene lead to decreased (p.M33T, p.D256N), increased (c.399 C > T) or to absent (p.Y242X) enzyme activity^4^. The presence of these changes has been described with different frequencies in Polish and other populations^14^. The substitution p.M33T was proved to determine the pharmacokinetic profile of propofol and the reduced catalytic efficiency of the enzyme^15^.

Sulfotransferase 1A1 (SULT1A1) and NAD(P)H quinone dehydrogenase 1 (NQO1) play a role in the second phase of propofol metabolism. Among the many polymorphic changes in *SULT1A1* (OMIM171150) and *NQO1* (OMIM125860) genes, two alleles have been subjected to particularly intense investigation: NQO1*2 (p.P187S) and SULT1A1*2 (p.R213H), both leading to decreased expression^16,17^.

Long-term studies on the P-glycoprotein 1 encoded by the *ABCB1* gene (OMIM171050), known as multidrug-resistance-protein 1, indicate the involvement of genetic polymorphism in the expression and activity of P-gp, which may affect the bioavailability, efficacy, and toxicity of drugs that are the substrates. Variants located in positions c.3435 and c.2677 of the *ABCB1* gene have been analyzed in the context of opioid pharmacokinetics. Potentially, they could also affect the PK of propofol^18^.

Investigations demonstrate, that variants of the serum albumin gene *ALB* (OMIM103600), may considerably affect the transport of certain drugs by albumin. The change located in exon 7, c.725 G > C (p.R218P) or c.725 G > A (p.R218H) causes decreased binding of warfarin to the albumin, altering the pharmacokinetics of the drug^19^. Moreover, disturbed binding of drugs to this protein also cause variations c.1011 G > T (p.K313N) and c.1165 G > C (p.D365H)^20^.

Because the mechanism of propofol action is based on its interaction with an ionotropic receptor GABA_A_, which inhibits the transfer of nerve impulses between neurons in the central nervous system, sequence changes of the *GABRA1* gene (OMIM 137160) may influence the effect of anaesthesia^21^. Stewart *et al*. proved the correlation between mutation p.M236W in the *GABRA1* gene and the influence of etomidat anaesthetic on the receptor^22^. Moreover, studies have shown that polymorphism of the adrenergic receptor gene (*ADRA1A*, OMIM104221) influences the expression of these proteins, the post-translational processes and pharmacological response by the receptor interfering with the signal transduction, what may change the regulation of a number of relevant parameters in anaesthesia^9^.

The aim of our study was to identify the genetic determinants of diverse pharmacokinetics and pharmacodynamics of propofol, using deep sequencing of 9 candidate genes including: *CYP2C9*, *CYP2B6*, *UGT1A9*, *SULT1A1*, *NQO1*, *ABCB1*, *ALB*, *GABRA1* and *ADRA1A*.

## Results

### Long range amplification and NGS

**Figure 1:**
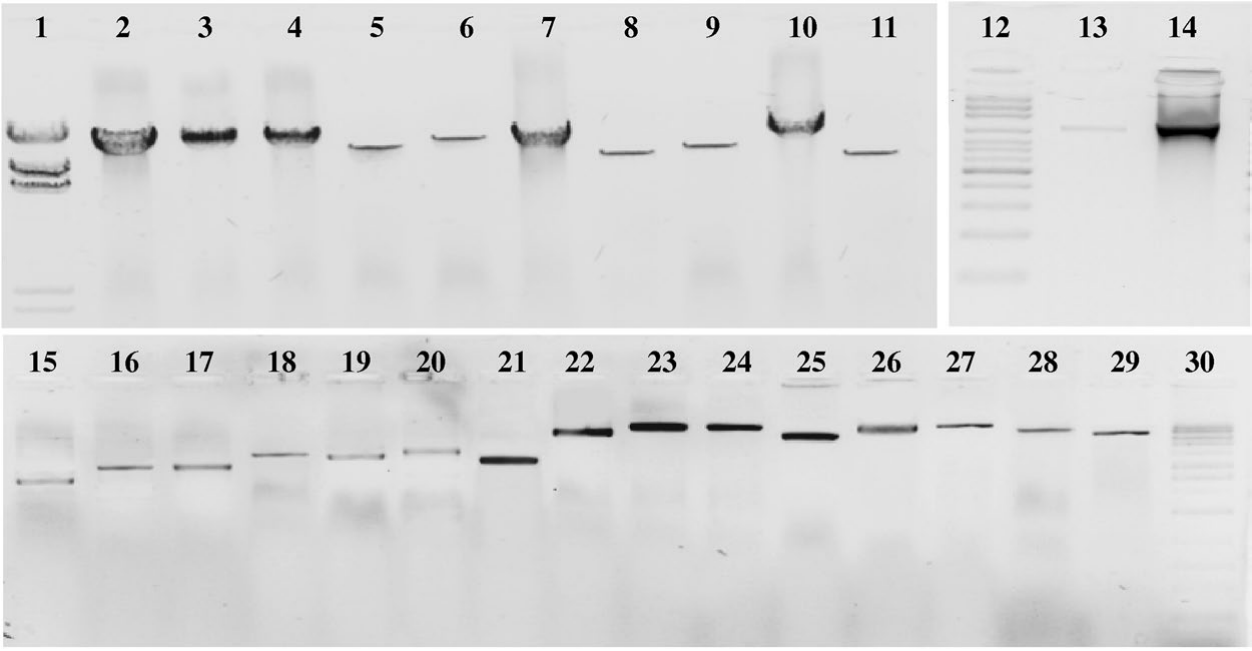
Results of 27 LR-PCR amplifications for one patient. Line 1 - ladder λ DNA *Hind*III; 2 - GABRA1 (17533 bp), 3 - ABCB1 (16809 bp); 4 - ABCB1 (17000 bp); 5 - NQO1 (11767 bp); 6 - ABCB1 (14483 bp); 7- ABCB1 (15827 bp); 8 - GABRA1 (10134 bp); 9 - ALB (11245 bp); 10 - CYP2C9 (17670 bp); 11 - CYP2C9 (10723 bp); 12 – ladder 100 bp; 13 - UGT1A9 (972 bp); 14 - ADRA1A (1183 bp); 15 - UGT1A9 (2303 bp); 16 - SULT1A1 (2760 bp); 17 - CYP2C9 (2761 bp); 18 - CYP2B6 (3312 bp); 19 - SULT1A1 (3219 bp); 20 - ABCB1 (3518 bp); 21 - CYP2B6 (3113 bp); 22 - ADRA1A (6818 bp); 23 - CYP2B6 (9280 bp); 24 - GABRA1 (9190 bp); 25 - ABCB1 (5967 bp); 26 - ALB (8571 bp); 27 - NQO1 (8998 bp); 28 - UGT1A9 (6700 bp); 29 - SULT1A1 (6497 bp); 30 – ladder 1 kbp;. Lines 1–11, 0.5% agarose gel; lines 12–14, 1.5% agarose gel; lines 15–30, 1.0% agarose gel. The gel images were obtained by trimming and colour adjusting of the full-length gels in the IrfanView 4.44 program.

We optimized all twenty-seven LR-PCRs using three sets of polymerases and successfully amplified over 226 kb of the DNA for 87 patients anaesthetized with propofol (Fig. 1 **)**. Full-length gels are presented in Figure S1.

**Figure 2:**
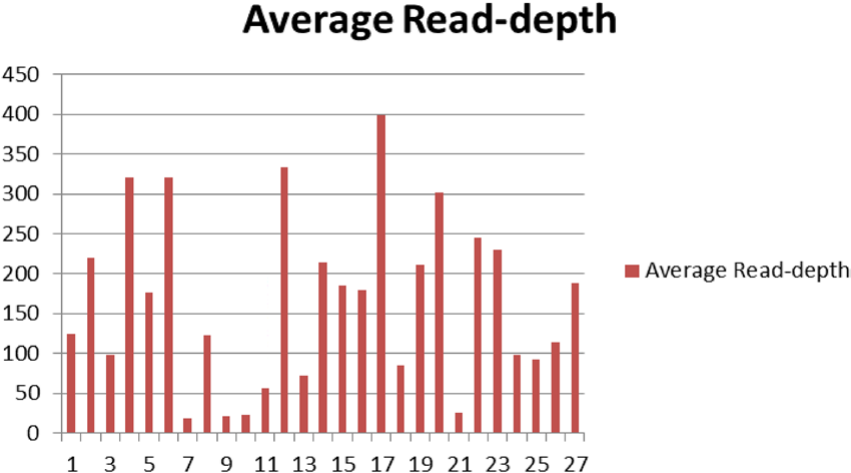
Mean read-depth of all amplicons in the NGS analysis.

All 87 DNA libraries with a mean fragment length between 300–500 bp were successfully sequenced on the Illumina MiSeq platform. On average 77.2% of the reads were aligned to the target regions. The mean depth of the reads was 166 (between 19–399) (Fig. 2). Four amplicons (numbered 7, 9, 10, 21) showed a considerable lower reads depth (<30-fold) among an average of 66% of the patient group analyzed and were excluded from further analyses. Moreover, amplicon number 11 showed insufficient coverage of approximately 25% of patients. Finally, only those variants of which the *locus* was read minimum 30 times in all patients, were included in the further analysis.

We detected 1513 different sequence variations in total. They were annotated and filtered using SNP-nexus and Variant Studio (Illumina). From the whole amount of the variants detected we selected only those described in the Human Genome Mutation Database (HGMD^®^Professional 2015.4) and nonsynonymous changes (Table 1). These changes were randomly verified with Sanger sequencing.

**Table 1:** Selected variants with potential pharmacokinetic significance.

No.	Number rs	Gene	Nucleotide change	Aminoacid change	Number HGMD^®^	Impact for drug response/ADR	Obtained allele frequency (%)	Allele frequency in Caucasians/European based on “1000 Genomes Project” (%)
1.	rs1057910	*CYP2C9*	c.1075 A > C	p.I359L	CM960481	Poor metabolism	4.6	2–10
2.	rs182132442		c.835 C > A	p.P279T	—	?	0.6	0–2
3.	rs28371674		c.430 C > T	p.R144C	CM994193	Poor metabolism	16.7	8–17
4.	rs4918758		−1188T > C	—	CR016116	Decreased enzyme activity	36	32–40
5.	rs3211371	*CYP2B6*	c.1459 C > T	p.R487C	—	Doxorubicin toxicity	9.2	9–14
6.	rs34883432		c.62 A > T	p.Q21L	—	?	0.6	<1
7.	rs35979566		c.1172 T > A	p.I391N	CM042695	Impaired enzyme activity	0.6	<1
8.	rs35303484		c.136 A > G	p.M46V	CM042692	Impaired enzyme activity	0.6	<1
9.	rs780991919		c.1061 A > G	p.Y354C	—	?	2.9	<1
10.	rs8192709		c.64 C > T	p.R22C	—	Cyclophosphamide toxicity	8	4–8
11.	rs749188589		c.1078 T > G	p.S360A	—	?	0.6	0–2
12.	rs45482602		c.777 C > A	p.S259R	—	?	0.6	<1
13.	rs35773040		c.419 G > A	p.R140Q	—	?	2.3	0–2
14.	rs58871670		c.547 G > A	p.V183I	—	?	1.2	0–2
15.	rs138030127		c.1021 C > G	p.H341D	—	?	2.9	0–2
16.	rs565104467		c.1016 A > C	p.E339A	—	?	2.3	0–2
17.	rs150072531		c.1190 A > G	p.H397R	—	?	0.6	<1
18.	rs56308434		c.516 G > T	p.Q172H	CM130453	Impaired metabolism of S-metadon	18.4	15–30
19.	rs2279343		c.785 A > G	p.K262R	CM034032	Impaired enzyme activity	19.5	11
20.	rs4803419		c.485–18 C > T	—	CS033987	Modified expression	36.8	27–37
21.	rs34223104		−82T > C	—	CR050427	Impaired expression	2.3	<1
22.	—	*ALB*	c.1226 A > G	p.Q409R	—	?	0.6	—
23.	rs2229109	*ABCB1*	c.1199 C > T	p.S400N	CM045736	Impaired transport	5.2	2–20
24.	rs2032582		c.2677 T > G	p.S893A	CM033585	?	27	21–43
25.	—		c.2687 T > C	p.I896T	—	?	0.6	—
26.	-		c.2602 A > G	p.I868V	—	?	0.6	—
27.	rs9282564		c.61 T > C	p.N21D	—	Morphine toxicity	5.2	5–10
28.	rs55852620		c.3320 T > G	p.Q1107P	—	?	0.6	<1
29.	rs1045642		c.3435 C > T	p.I1145=	CM000496	Decreased protein level	43.7	37–53
30.	rs1128503		c.1236 A > G	p.G412=	CM084696	Sunitinib toxicity	46	50–60
31.	rs2229125	*ADRA1A*	c.599 A > C	p.I200S	CM057865	Impaired binding of antagonist	2.3	0–2
32.	rs1048101		c.1039 A > G	p.C347R	CM064954	Nifedipine eficacy	47.1	37–47
33.	rs1800566	*NQO1*	c.559 C > T	p.P187S	CM950861	Benzene toxicity	20.7	18–25
34.	rs1131341		c.415 C > T	p.R139W	CS024267	Decreased enzyme activity	4.6	2–3.5
35.	rs689455		−1128T > G	—	CR087455	Decreased transcription level	19	18–25
36.	rs72551330	*UGT1A9*	c.98 T > C	p.M33T	CM033677	Decreased enzyme activity	2.3	1.5–2.5
37.	rs9282861	*SULT1A1*	c.638 G > A	p.R213H	CM973382	Decreased enzyme activity	27	22–30

### Pharmacokinetics and pharmacodynamics of propofol

The mean value of MRT was 82.5 min and the range was 8.0–504.1 min (SD = 107 min). Based on a percentile rank we determined three pharmacokinetic profiles of the propofol metabolism rate in our group of patients: rapid (MRT ≤ 30 min), intermediate (100 ≥ MRT > 30 min) and poor (MRT > 100 min) metabolizers, who constitute 27%, 48% and 22% of the group, respectively.

**Figure 3:**
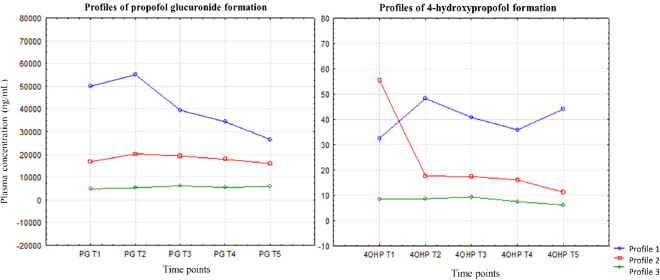
Designated profiles of PG and 4-OHP formation. Mean plasma concentrations of PG and 4-OHP measured directly after finished propofol administration (T1), 5 min (T2), 10 min (T3), 20 min (T4) and 30 min (T5) after.

To specify the differences in the biotransformation pathway of propofol we analyzed the formation profile of two main metabolites: 4-hydroxypropofol (4-OHP) and propofol glucuronide (PG). Using a k-means clustering, we determined three significantly different (P = 0.00) groups of patients due to the formation of PG and 4-OHP (Fig. 3).

Profile 1 of PG formation was observed in 13 of patients, profile 2 and 3 in 23 and 44 individuals, respectively. Clearly, 4-OHP was produced to a minor extent, relative to PG, the second metabolite. Profile 1 of 4-OHP formation was reported in 14 of patients, profile 2 in 13 of the subjects and profile 3 was presented by 53 individuals (Fig. 3).

The pharmacodynamics of the anaesthetic were described by the awakening time of the patients after propofol anaesthesia. The mean awakening time was 12 min, the range was 0.5–45 min (SD = 7.8 min).

### Correlation of genetic variants with PK and PD of propofol

The impact of genetic background on the pharmacokinetics of propofol was proved by correlation analysis of genetic changes found in genes coding for metabolizing and in transporting proteins of propofol with MRT and formation profiles of PG and 4-OHP. The statistical significance of distribution differences of selected variants in rapid, intermediate and poor metabolizers is shown in Table 2.

**Table 2:** Genetic variants distribution in different profiles of propofol metabolism rate.

Gene	Change	Genotype	Intermediate metaboliser	Rapid metaboliser	Poor metaboliser	Statistical significance (P-value)
*CYP2C9*	c.1075 A > C p.I359L	AA	40	21	18	0.747
		AC	3	3	2	
		CC	0	0	0	
	c.835 C > A p.P279T	CC	42	24	20	0.595
		CA	1	0	0	
		AA	0	0	0	
	c.430 C > T p.R144C	CC	28	18	12	0.549
		CT	15	6	8	
		TT	0	0	0	
	−1188T > C	TT	17	14	7	0.094
		TC	25	7	10	
		CC	1	3	3	
*CYP2B6*	c.1459 C > T p.R487C	CC	34	20	17	0.824
		CT	9	4	3	
		TT	0	0	0	
	c.62 A > T p.Q21L	AA	43	24	19	0.183
		AT	0	0	1	
		TT	0	0	0	
	c.1172 T > A p.I391N	TT	43	24	19	0.183
		TA	0	0	1	
		AA	0	0	0	
	c.136 A > G p.M46V	AA	42	24	20	0.595
		AG	1	0	0	
		GG	0	0	0	
	c.1061 A > G p.Y354C	AA	42	21	19	0.226
		AG	1	3	1	
		GG	0	0	0	
	c.64 C > T p.R22C	CC	38	21	14	0.154
		CT	5	3	6	
		TT	0	0	0	
	c.1078 T > G p.S360A	TT	43	24	19	0.183
		TG	0	0	1	
		GG	0	0	0	
	c.777 C > A p.S259R	CC	43	23	20	0.265
		CA	0	1	0	
		AA	0	0	0	
	c.419 G > A p.R140Q	GG	41	23	19	0.991
		GA	2	1	1	
		AA	0	0	0	
	c.547 G > A p.V183I	GG	41	24	20	0.350
		GA	2	0	0	
		AA	0	0	0	
	c.1021 C > G p.H341D	CC	39	24	19	0.288
		CG	4	0	1	
		GG	0	0	0	
	c.1016 A > C p.E339A	AA	40	24	19	0.423
		AC	3	0	1	
		CC	0	0	0	
	c.1190 A > G p.H397R	AA	42	24	20	0.595
		AG	1	0	0	
		GG	0	0	0	
	c.516 G > T p.Q172H	GG	28	14	16	0.140
		GT	15	7	4	
		TT	0	3	0	
	c.785 A > G p.K262R	AA	27	17	16	0.328
		AG	14	4	3	
		GG	2	3	1	
	c.485–18 C > T	CC	17	10	6	0.910
		CT	21	11	12	
		TT	5	3	2	
	−82T > C	TT	41	23	19	0.991
		TC	2	1	1	
		CC	0	0	0	
*ALB*	c.1226 A > G p.Q409R	AA	43	24	19	0.183
		AG	0	0	1	
		GG	0	0	0	
*ABCB1*	c.1199 C > T p.S400N	CC	40	21	17	0.573
		CT	3	3	3	
		TT	0	0	0	
	c.2677 T > G p.S893A	TT	25	18	10	0.010
		TG	14	1	6	
		GG	4	5	4	
	c.2687 T > C p.I896T	TT	43	23	20	0.265
		TC	0	1	0	
		CC	0	0	0	
	c.2602 A > G p.I868V	AA	42	24	20	0.595
		AG	1	0	0	
		GG	0	0	0	
	c.61 T > C p.N21D	TT	38	22	19	0.398
		TC	5	1	1	
		CC	0	1	0	
	c.3320 T > G p.Q1107P	TT	43	23	20	0.265
		TG	0	1	0	
		GG	0	0	0	
	c.3435 C > T p.I1145=	CC	16	8	7	0.400
		CT	20	8	6	
		TT	7	8	7	
	c.1236 A > G p.G412=	AA	14	9	7	0.907
		AG	19	8	7	
		GG	10	7	6	
*NQO1*	c.559 C > T p.P187S	CC	30	13	13	0.789
		CT	11	9	6	
		TT	2	2	1	
	c.415 C > T p.R139W	CC	40	22	17	0.582
		CT	3	2	3	
		TT	0	0	0	
	−1128T > G	TT	30	15	13	0.974
		TG	11	8	6	
		GG	2	1	1	
*UGT1A9*	c.98 T > C p.M33T	TT	41	24	18	0.288
		TC	2	0	2	
		CC	0	0	0	
*SULT1A1*	c.638 G > A p.R213H	GG	21	10	13	0.465
		GA	19	13	7	
		AA	3	1	0	

Two genetic changes initially have shown a correlation with the propofol metabolic profile, c.516 G > T (p.Q172H) in *CYP2B6* gene and c.2677 T > G (p.S893A) in *ABCB1* gene. Homozygotes c.516 T/T were more often (P = 0.046) presented in the group of rapid metabolizers, while heterozygotes c.2677 T/G were more frequently (P = 0.032) observed in patients presenting an intermediate and poor metabolism rate of the anaesthetic. However, using Bonferroni correction, the P-values were not statistically significant (P = 0.14, P = 0.1 for c.516 G > T and c.2677 T > G, respectively).

Moreover, we found that mutation p.S400N (c.1199 C > T) in the *ABCB1* gene has a substantial impact on the profile of PG formation in our anaesthetized patient group. Homozygotes c.1199 C/C determined the profile 2 of the PG formation rate. However, none of variants analyzed affected the rate of 4-OHP formation.

The pharmacodynamics of propofol was correlated with genetic changes located in gene coding for the adrenergic receptor. The statistical significance of the influence of *ADRA1A* gene variants for the recovery time after anaesthesia is presented in Table 3. These analyses revealed no correlation between the pharmacodynamics of propofol and selected variants in the *ADRA1A* gene.

**Table 3:** Genetic variants distribution in different recovery time groups.

Gene	Change	Genotype	Recovery time			Statistical significance(P-value)
			0–10 min (%)	10–20 min (%)	>20 min (%)	
*ADRA1A*	c.599 A > C p.I200S	AA	98	92	100	0.472
		AC	2	4	0	
		CC	0	4	0	
	c.1039 A > G p.C347R	AA	35	13	30	0.109
		AG	53	54	40	
		GG	12	33	30	

## Discussion

Personalized anaesthesia with propofol still remains a challenge for pharmacogenomics. Global studies indicate in particular the contribution of *CYP2B6*, *CYP2C9* and *UGT1A9* gene polymorphism to the variability of propofol pharmacokinetics and pharmacodynamics^6–8,23^. Considering the complexity of the propofol biotransformation pathway, transport and interactions with receptors, in this study we presented deep sequence analysis of 9 gene panels, including those coding for receptors (*GABRA1*, *ADRA1A*), transporters (*ALB*, *ABCB1*) and liver enzymes (*UGT1A9*, *CYP2B6*, *CYP2C9*, *NQO1*, *SULT1A1*) in the context of anaesthetic metabolism rate, recovery time and searching for genetic determinants of individual propofol response.

Our investigations softly suggested that of the all genes analyzed, *CYP2B6* and *ABCB1* polymorphism may possibly influence propofol pharmacokinetics in this Polish patients group, what should be an object of further investigation. This finding confirms global reports and our previous results, proving the importance of variant p.Q172H (c.516 G > T; CM130453) in *CYP2B6* gene for the propofol metabolism rate^24^. In our study homozygous c.516 T/T was identified more often in the rapid metabolizers of propofol, but no statistically significant correlation was found, in the contrast to Kansaku *et al*.^7^ and Mastrogianni *et al*.^8^, whose observations proved an association of a surprisingly higher propofol concentration in the serum with allele T^7,8^. On the other hand, Loryan *et al*.^23^ and Khan *et al*.^25^ did not find any correlation between polymorphism p.Q172H and the plasma concentration of propofol or its metabolites^23,25^. We also cannot share Murao *et al*.‘s (2015) conclusions that allele T decreased the total required dose of propofol^6^. The results of these different studies are contradictory, however, they do confirm the importance of a particular gene and a defined *locus*.

Moreover, we observed that heterozygotes c.2677 T/G of substitution p.S893A (c.2677 T > G; CM033585) located in exon 21 of the *ABCB1* gene were more frequent (but not significantly) in the group of intermediate- and poor- propofol metabolizers. So far, there has been a lack of investigations that would analyse changes in the *ABCB1* gene in the context of the reaction and the biotransformation rate of propofol. Nevertheless, the *ABCB1* gene is considered to be one of the most important pharmacogenes and the impact of polymorphic variability is widely examined in the context of a number of therapies, especially of lymphocytic leukemia^26^. The change p.S893A resides in an important region between the membrane and the cytosolic N-terminal NBD in the part of TM10 of the ABCB1 protein^27^.

As evidence of the variable biotransformation process of propofol, in our experimental group we identified considerable interindividual differences in the formation of propofol metabolites, both PG and 4-OHP. Interestingly, as a causal genetic factor linked to interpatient variability in the formation rate of PG, we found the mutation p.S400N (c.1199 C > T) in the *ABCB1* gene. Homozygous individuals c.1199 C/C revealed the profile 2 (“middle homogeneous”) of the glucuronidation rate. This mutation (CM045736) is known as disruptive of the ABCB1 protein transport activity and therefore it can probably influence propofol disposition and anaesthesia efficiency^28^.

The concept for molecular analysis in our investigations consisted of applying an approach combining LR-PCR with NGS, which enabled us to conduct an in-depth analysis of over 20 Mb of the sequence in a single run. A great advantage of using amplicon libraries and such methodology, compared to commercial sets of probes, is the easy and inexpensive modification of targeted genetic fragments through changes at the stage of designing long-range PCR. So far, only a few studies exist worldwide, which used a similar solution successfully^29–31^. On the other hand, we are of course aware of the limitations of our study, such as the number of patients, possible interactions between propofol with other drugs and the analysis of NGS data. It was crucial for us to obtain sufficient coverage for the genomic regions analyzed, which we unfortunately failed to achieve for a few amplicons, therefore 4 of them, covering exons 3–6 of the *SULT1A1* gene, promoter and exons 1–8 of the *GABRA1* gene and exons 6–15 of the *ALB* gene, were excluded from the final analysis. We suspect that they also can contain important changes for propofol action. To improve the experiment, perhaps the LR-PCR products of these 4 fragments should be added to the library in higher quantitative ratios in relation to other fragments.

Because we obtained a large amount of NGS data as detected variants, we decided to limit our analysis in the first stage only to the genetic changes recognized as most important from the pharmacogenetic point of view (mutations described in the HGMD database and as yet unknown amino acid changes). Nevertheless, our investigations are the first such a wide genetic analysis of the pharmacokinetics and pharmacodynamics of propofol in the world and may potentially allow genes to be identified that may play an important role in the interindividual variability of its anaesthetic action.

## Materials and Methods

### Patients and samples

Eighty-seven Polish patients (34 female and 53 men), Caucasians undergoing general anaesthesia with propofol (10 mg/mL propofol injectable emulsion, Diprivan; AstraZeneca, Macclesfield, UK) before minor laryngological surgery at the Regional Hospital in Poznan, Poland, were enrolled in this study. All participants provided written informed consent. The study was approved by the local Ethics Committee of the University of Medical Sciences in Poznan, Poland (resolution no. 653/09) and all experiments were performed in accordance with relevant guidelines and regulations of this Committee. Detailed information about the patients: age, weight, height, dose of propofol, infusion time, awakening time and adverse effects were collected (Table 4). All patients represented I or II class on The American Society of Anesthesiologists (ASA) scale. Perioperative monitoring includes heart rate, blood pressure and saturation. Anaesthesia was induced with propofol (2 mg/kg) followed by continuous infusion at a rate of 8 mg/kg/h, as described previously^23^. From each patient, 5 mL of peripheral blood for molecular analysis and 2 mL of blood samples were taken at 5 time points as follows: at the end of anaesthesia, 5, 10, 20 and 30 minutes after. These samples were collected for pharmacokinetic study.

**Table 4:** Characteristics of patients.

Parameter	Mean	Range
Age	44.4	31–53
BMI	27.0	19.2–44.8
Total dose of propofol (mg)	692	130–2,200
Infusion time (min)	47	10–145
Awakening time (min)	12	0.5–45
Observed side effects (strong pain after anaesthesia)	2 patients	—

### LR-PCR amplification

The genomic DNA of each patient was isolated from the peripheral blood samples using the method with guanidine isothiocyanate (GTC). Detection of genetic variants in the coding sequence, including splice junctions and promoter regions of nine genes: *UGT1A9*, *CYP2B6*, *CYP2C9*, *SULT1A1*, *NQO1*, *GABRA1*, *ADRA1A*, *ALB* and *ABCB1* was performed by NGS on MiSeq^®^ System (Illumina, San Diego, USA). Amplicon libraries for NGS analysis were prepared based on twenty-seven LR-PCR fragments. Pairs of primer sets were designed in reference to the human genomic sequence (GRCh37/hg19) using Primer Blast and Primer 3 Plus software. In total, over 226 kb of the DNA of each patient were amplified in fragments ranging between 1 and 18 kbp (Table S1).

After optimization, LR-PCR reactions were carried out on an Applied Biosystems 2720 Thermal Cycler (Applied Biosystems, Foster City, CA) with the use of one of three polymerase sets: GoTaq^®^ Long PCR Master Mix (Promega, Madison, USA), Long & High Fidelity PCR Enzyme Mix (BiotechRabbit, Hennigsdorf, Germany), Fire Pol^®^DNA Polymerase (Solis BioDyne, Tartu, Estonia) on the total volume of 30 μL, according to the manufacturer’s instructions (Table 5). Generally, most of the fragments were amplified using the GoTaq^®^ Long PCR Master Mix (Promega) polymerase in accordance with the standard 2-step or 3-step PCR program.

**Table 5:** Conditions of LR-PCR reactions.

Polymerase	Amplified fragments	Reaction mixture	PCR program
Promega (GoTaq^®^ Long PCR Master Mix)	1, 3, 7, 10, 13, 15, 18, 20	58 ng template DNA, 0.72 μL of a 5 μmol/L primers, 15 μL of master mix water up to 30 μL	94 °C 2 min, 35 cycles, 94 °C 30 sec, 65 °C 1 min/kb, 72 °C 10 min, Hold at 4 °C
	4, 5, 8, 9, 11, 16, 17, 19, 21, 22, 23, 24, 25, 26, 27	58 ng template DNA, 0.72 μL of a 5 μmol/L primers, 15 μL of master mix, water up to 30 μL	94 °C 2 min, 35 cycles, 94 °C 30 sec, 62 °C 30 sec, 65 °C 1 min/kb, 72 °C 10 min, Hold at 4 °C
BiotechRabbit (Long & High Fidelity PCR Enzyme Mix)	6, 12, 14	40 ng template DNA, 1.2 μL of a 5 μmol/L primers, 3 μL 10x buffer, 3 μL dNTPs, 0.3 μL polymerase, 1.8 μL 25 mM MgCl_2_, 5 μL of Helper (frag. 12), 2.1 μL of Taq-stabiliser (frag. 6), 6 μL of GC-enhancer (frag. 6), water up to 30 μL	95 °C 2 min, 35 cycles, 95 °C 30 sec, 65 °C, −0.5°/cycle 45 sec, 68 °C 1 min/kb, 72 °C 5 min, Hold at 4 °C
Solis BioDyne (Fire Pol^®^DNA Polymerase)	2	80 ng template DNA, 1.2 μL of a 5 μmol/L primers, 3 μL 10x buffer, 2.4 μL dNTP, 1.8 μL 25 mM MgCl_2_, 0.18 μL polymerase, water up to 30 μL	95 °C 5 min, 35 cycles, 95 °C 45 sec, 56 °C 45 sec, 72 °C 1 min, 72 °C 5 min, Hold at 4 °C

### NGS Library Preparation

After visualization on the agarose gel, the LR-PCR fragments obtained were purified with Exo-Sap (Affymetrix, Santa Clara, USA) before they were quantified using Qubit dsDNA BR Assay System (Invitrogen, Carlsbad, USA) on the Qubit® 2.0 Fluorometer (Thermo Fisher, Waltham, USA).

In the next step, 27 amplicons of each patient were pooled in equimolar ratios. According to the manufacturer’s protocol, 1 ng of the pooled DNA fragments was subjected to the Nextera XT procedure (Illumina) using transposome technology. Finally, using Nextera XT DNA Sample Preparation Kit (Illumina) and Nextera^®^ XT Index Kit (96) (Illumina), we obtained eighty-seven both-side indexed DNA libraries ready for high-throughput sequencing. Quality control of the libraries was performed on TapeStation 2200 Instrument (Agilent, Santa Clara, USA) using an HS D1000 ScreenTape (Agilent). Normalization of all libraries was carried out with magnetic beads, according to the Nextera XT procedure.

### Sequencing and bioinformatic analysis

Sequencing on the Illumina MiSeq System was performed as paired-end targeted resequencing using a MiSeq Reagent Kit v2 (300 cycle) (Illumina). Obtained reads were mapped to the reference DNA sequence (GRCh37/hg19) by a Burrows-Wheeler Aligner (BWA, version v0.7.10-r789) algorithm with default settings. Then, variants located in regions involved in the manifest were inspected using IGV (Integrative Genomics Viewer, version v2.3.59). The changes detected were then filtered using a set of criteria: GQX (genotyping quality) ≥ 30, read depth ≥ 30 and heterozygous read ratio ≥ 35%. Variants with potential pharmacokinetic significance included in Table 1 were randomly confirmed using Sanger sequencing. Amplicons were purified using shrimp alkaline phosphatase and exonuclease I, following manufacturer’s instructions. Direct sequencing was performed using BigDye Terminator v3.1 Cycle Sequencing Kit (Thermo Fisher Scientific) on the Applied Biosystems 3500 and Series Genetic Analyzers. Primer sequences and PCR conditions are available upon request.

### HPLC measurements

The propofol concentration, as described previously^24^, as well as its two main metabolites: 4-hydroxypropofol and propofol glucuronide (Fluorochem Ltd., UK) in plasma retrieved from patients at five different time points was measured using the HPLC/UV (P580A, Dionex, Germany) system coupled to a fluorescence (RF2000, Dionex) detector. Plasma samples (150 μL) were mixed with 150 μL of 2 M trichloroacetic acid (TCA) and certifugated at 10,000 g for 10 min. An aliquot of the supernatant was injected onto an analytical C18 reversed-phase column (Hypersil GOLD, 250 mm × 4.6 mm × 5 µm, Thermo Fisher Scientific, USA) maintained at 30 °C. The mobile phase constituted 0.6% (v/v) orthophosphoric (V) acid and acetonitrile (50:50) at a flow rate of 1.0 ml/min. The elution profiles of propofol and 4-hydroxypropofol were monitored fluorometrically at an excitation wavelength of 270 nm and an emission wavelength of 310 nm and propofol glucuronide by HPLC/UV at wavelength of 260 nm. The plasma concentrations of propofol and its metabolites were determined by Chromeleon software version 6.80 (Dionex). For each analysis the percentage of relative standard deviation (RSD) was calculated and for the HPLC/UV and fluorescence method was below 2.5%. All samples were analyzed in duplicate.

### Pharmacokinetic and pharmacodynamic parameters of propofol

The pharmacokinetics of propofol was characterized by the mean removal time (MRT), determined using a PKSolver tool based on HPLC measurements of the propofol concentration, dose of anaesthetic and infusion time^32^. Moreover, for both metabolites (propofol glucuronide and 4-hydroxypropofol) different profiles of the formation rate were defined by clustering analysis using k-means. Awakening time, characterized as the mean time of eye opening, first breath and orientation were used as pharmacodynamic parameter of propofol anaesthesia.

### Statistical analysis

Analysis of the correlation between DNA sequence variants, MRT results, metabolites profiles and awakening time values was performed using Chi2 with Fisher’s test corrections. For multiple testing Bonferroni corrections were used. For all calculations STATISTICA 12.0 software (Stat Soft, 2014) was used. A P-value lower than 0.05 was considered statistically significant.

## Electronic supplementary material

Supplementary Information
