# Supplementary material for: Longrange PCR-based next-generation sequencing in pharmacokinetics and pharmacodynamics study of propofol among patients under general anaesthesia
Source: Sci Rep. 2017 Nov 13;7:15399. doi: 10.1038/s41598-017-15657-2 (PMC5684313; doi:10.1038/s41598-017-15657-2)
Supplement: Supplementary file 1 — Supplementary Information [file 41598_2017_15657_MOESM1_ESM.doc]

**Long-range PCR-based next-generation sequencing in pharmacokinetics and pharmacodynamics study of propofol among patients under general anaesthesia.**

**Running title:** Next-generation sequencing in propofol study.

Oliwia Zakerska-Banaszak, Marzena Skrzypczak-Zielinska, Barbara Tamowicz, Adam Mikstacki, Michal Walczak, Michal Prendecki, Jolanta Dorszewska, Agnieszka Pollak, Urszula Lechowicz, Monika Oldak, Kinga Huminska, Marta Molinska-Glura, Marlena Szalata, Ryszard Slomski


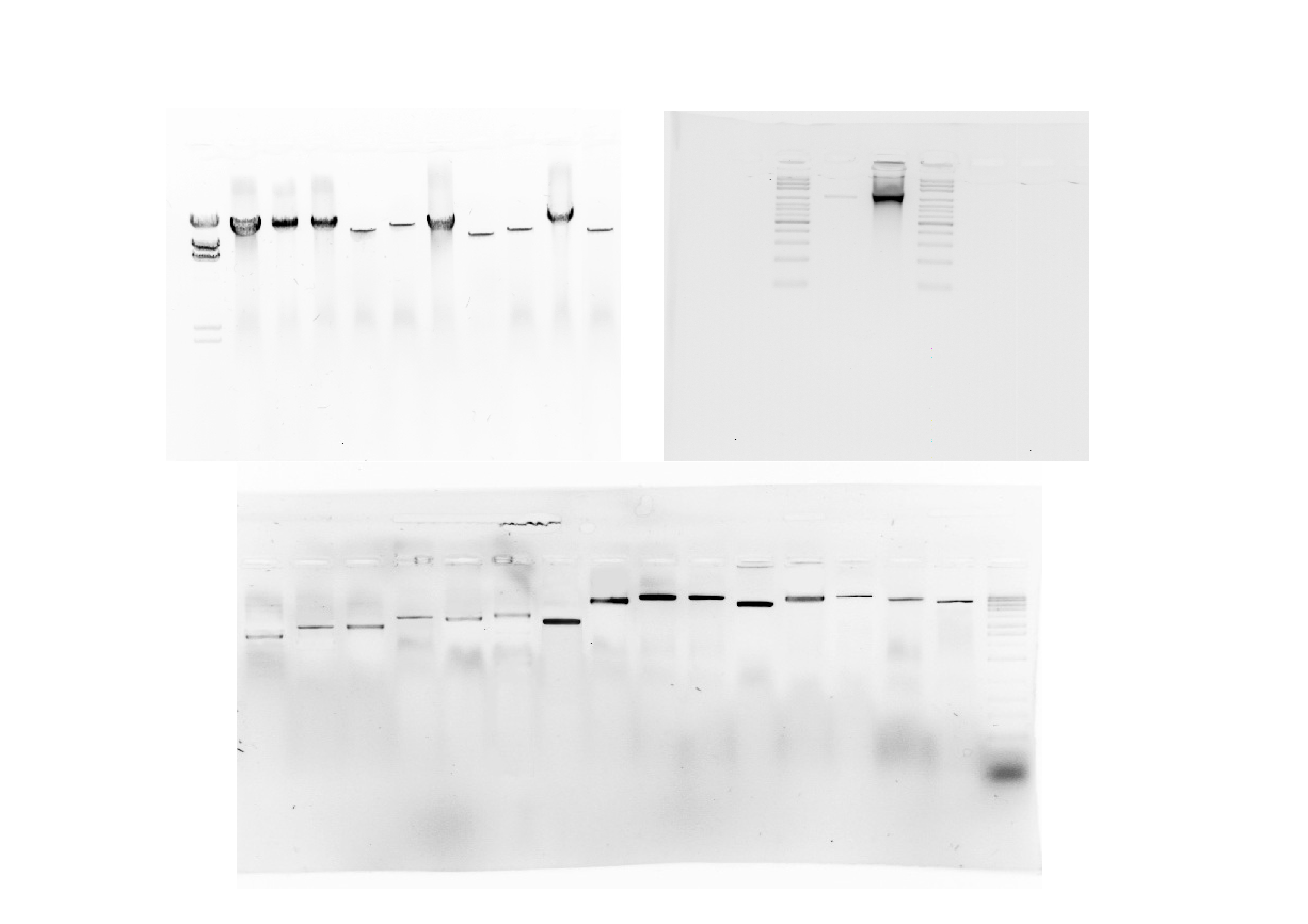


**Figure S1**. Full-length gels of results of 27 LR-PCR amplifications for one patient.

**Table S1. Genomic regions for** the NGS analysis.

| **Fragment** | **Amplified region** |  | **Primer sequence 5’→3’** | **Tm**  **(°C)** | **Amplicon**  **length**  **(bp)** | **Chromosome** |
| --- | --- | --- | --- | --- | --- | --- |
| 1 | *UGT1A9*_Promoter | For | TTGAGACAGAGTCGTGCTGTTT | 53.0 | 2303 | chr2 |
|  |  | Rev | GCAAAGCCACAGGTCAGC | 52.6 |  |  |
| 2 | *UGT1A9*_Ex1 | For | TGGTATTTCTCCCACCTACT | 53.7 | 972 |  |
|  |  | Rev | CCAAAGGTGAAGTATTCTTA | 49.7 |  |  |
| 3 | *UGT1A9*_Ex 2-5 | For | CTTCCATATCATTTAAAGGGACCACG | 56.4 | 6700 |  |
|  |  | Rev | GAAATAAATGCGACACCAGTGAAAAC | 54.8 |  |  |
| 4 | *NQO1*_Promoter-Ex2 | For | TCAGTTTTTGCCCTTATTTAATCCCC | 54.8 | 11767 | chr16 |
|  |  | Rev | AACTAATTAAAGAGGGGAGGAGGAAC | 56.4 |  |  |
| 5 | *NQO1*_Ex3-6 | For | CCTCCCCTCTTTAATTAGTTCTTTGC | 56.4 | 8998 |  |
|  |  | Rev | TCCCACTAGATTCCACTATCATTAGC | 56.4 |  |  |
| 6 | *SULT1A1*_Promoter-Ex2 | For | TTGGGCTGATGAACCATATTACAGT | 54.4 | 6497 | chr16 |
|  |  | Rev | TTTCCAATTCATGAGAAGTATAGACAC | 53.7 |  |  |
| 7 | *SULT1A1*_Ex3-6 | For | TGTCCTTGTTCTTTCTTGTTCTATGC | 54.8 | 3219 |  |
|  |  | Rev | GTCTACACTGAAGAGTCTTACGTTCT | 56.4 |  |  |
| 8 | *SULT1A1*_Ex7-10 | For | CAGTGTAGACCTAAGAAAGCTAGAGG | 58.0 | 2760 |  |
|  |  | Rev | CTTGGAATAGAGACTCTGCATTGAAC | 56.4 |  |  |
| 9 | *GABRA1*_Promoter-Ex4 | For | ATACAGAGGGATTAGAAAAGTGGGAC | 56.4 | 10134 | chr5 |
|  |  | Rev | AAACATACCAGTACCAGGTGAGATAG | 56.4 |  |  |
| 10 | *GABRA1*_Ex5-8 | For | ATCATTTAGGCAAGTATGTCCACTCT | 54.8 | 17533 |  |
|  |  | Rev | ATAGCTACCTGTTGTGCTACAGTAAT | 54.8 |  |  |
| 11 | *GABRA1*_Ex9-11 | For | AGTCAAATTGCTCATCTTTCTTGTGT | 53.2 | 9190 |  |
|  |  | Rev | CTTGGTACTTTCAGGTGCTTCTAATG | 56.4 |  |  |
| 12 | *ADRA1A*_Promoter-Ex2 | For | CTATGCGAGAGCAAAGTCTTATTGTT | 54.8 | 6818 | chr8 |
|  |  | Rev | ACCAATTAATCTGCATCAGTTGGAAG | 54.8 |  |  |
| 13 | *ADRA1A*_Ex3 | For | TTTGAAGATATTGCAAAAGGGTGACA | 53.2 | 1183 |  |
|  |  | Rev | CACCATCTTAATGCTCTTCCTCTCTA | 56.4 |  |  |
| 14 | *ABCB1*_Promoter-Ex1 | For | CAGTTCAGAAACAGAACAGAACATGA | 54.8 | 3518 | chr7 |
|  |  | Rev | AATGGCTTTGGAACTCTTAAGAAAGG | 54.8 |  |  |
| 15 | *ABCB1*_Ex2-5 | For | TTAGGAAGCAGAAAGGTGATACAGAA | 54.8 | 15827 |  |
|  |  | Rev | CTTGTTTTTGCTGCAAGTTTCCAATA | 53.2 |  |  |
| 16 | *ABCB1*_Ex6-10 | For | GATATTTCTGCTGACAGACTACCTCT | 56.4  54.8 | 16809 |  |
|  |  | Rev | TTCTAAAGTCAAGCCAACATTACTGG |  |  |  |
| 17 | *ABCB1*_Ex11-21 | For | CTGATGGTTTTTCTTCACATTCCTCA | 54.8 | 14483 |  |
|  |  | Rev | AAGGAGAAAATTAGTTTCATGCTGGG | 54.8 |  |  |
| 18 | *ABCB1*_Ex22-26 | For | TCATTCCTAGTTTGTCAGACTCCTTT | 54.8 | 17000 |  |
|  |  | Rev | CTCTCTATACCATCTGTCATTGCTGA | 56.4 |  |  |
| 19 | *ABCB1*_Ex27-29 | For | TAATTGTGCTACATTCAAAGTGTGCT | 53.2 | 5967 |  |
|  |  | Rev | ACCCCACCTCCTAAAATCTTATATCG | 56.4 |  |  |
| 20 | *ALB*_Promoter-Ex5 | For | AGATGGCATAAAAGCTGGACTAAATG | 54.8 | 8571 | chr4 |
|  |  | Rev | TTAAACACATGCACAGTGATTACCTC | 54.8 |  |  |
| 21 | *ALB*_Ex 6-15 | For | GGAGGGGTGTTTCATGTAGAATTTTT | 54.8 | 11245 |  |
|  |  | Rev | CCTTACCTATACCATGGTTTTTGTTCT | 55.2 |  |  |
| 22 | *CYP2C9*_Promoter | For | AATCTGAAAAACTGGGATTCTAAGA | 53.8 | 2761 | chr10 |
|  |  | Rev | GAAGGAGCATACTTACATTGGTT | 52.4 |  |  |
| 23 | *CYP2C9*_Ex1-5 | For | GCTTGGAGTGCAAGCTCATG | 53.8 | 10723 |  |
|  |  | Rev | GTAAACACAGAACTAGTCAAC | 52.4 |  |  |
| 24 | *CYP2C9*_Ex6-9 | For | TGGAATTTTTAGGCAAGCATGGAATA | 53.2 | 17670 |  |
|  |  | Rev | ATCCTCAAGTAACTCTAACACTCACC | 56.4 |  |  |
| 25 | *CYP2B6*_Promoter-Ex1 | For | GAAAGAGACTGGCTGAATGGA | 52.4 | 3312 | chr19 |
|  |  | Rev | TCCGTACTCACCAACCATGC | 53.8 |  |  |
| 26 | *CYP2B6*_Ex2-4 | For | ATGCTGACTAACAGCCACCC | 53.8 | 3113 |  |
|  |  | Rev | CCTGACCTGGCCGAATACAG | 55.9 |  |  |
| 27 | *CYP2B6*_Ex5-9 | For | AAACCTCACCACCCCTTCTTTCTT | 55.7 | 9280 |  |
|  |  | Rev | ACATTCCTTCCAGAAAAATCCCTAGA | 54.8 |  |  |
